# Diagnostic accuracy and cost-effectiveness of a handheld ultrasound device for cardiac evaluation in noncardiology settings

**DOI:** 10.1093/ehjimp/qyag047

**Published:** 2026-03-13

**Authors:** Stella-Lida Papadopoulou, Foteini Malakoudi, Christina Tsantekidou, Anastasios Papanastasiou, Dimitrios Dionysopoulos, Nikolas Moustakidis, Theofilos Moustakidis, Panagiotis Stafylas, Ioannis Styliadis, Petros Nihoyannopoulos, Areti Triantafyllou, Vasileios Sachpekidis

**Affiliations:** Department of Cardiology, Papageorgiou General Hospital, Ring Road, Nea Efkarpia, Thessaloniki 56403, Greece; Department of Cardiology, Papageorgiou General Hospital, Ring Road, Nea Efkarpia, Thessaloniki 56403, Greece; Department of Cardiology, Papageorgiou General Hospital, Ring Road, Nea Efkarpia, Thessaloniki 56403, Greece; Department of Cardiology, Papageorgiou General Hospital, Ring Road, Nea Efkarpia, Thessaloniki 56403, Greece; Department of Medical Oncology, School of Health Sciences, Faculty of Medicine, Papageorgiou Hospital, Aristotle University of Thessaloniki, Thessaloniki, Greece; Department of Informatics, Aristotle University of Thessaloniki, Thessaloniki, Greece; Echonous Inc., 8310 154th Ave NE, Redmond, WA 98052, USA; Echonous Inc., 8310 154th Ave NE, Redmond, WA 98052, USA; Department of Biomedical Informatics, University of Thessaly, Lamia, Greece; HealThink, 47 St.Kazantzidi str., PO Box 8121, Thessaloniki, Greece; Department of Cardiology, Papageorgiou General Hospital, Ring Road, Nea Efkarpia, Thessaloniki 56403, Greece; NHLI Hammersmith Hospital, Imperial College London, Du Cane Road, London W12 0NN, UK; First Propaedeutic Department of Internal Medicine, School of Health Sciences, Faculty of Medicine, AHEPA University Hospital, Aristotle University of Thessaloniki, Thessaloniki, Greece; Department of Cardiology, Papageorgiou General Hospital, Ring Road, Nea Efkarpia, Thessaloniki 56403, Greece

**Keywords:** consultative cardiology, point of care, echocardiography, handheld ultrasound devices

## Abstract

**Aims:**

Inpatients frequently require echo evaluation during consultation rounds in noncardiology departments; however, routine standard echocardiography (SE) requires transport to echo lab. We evaluated the diagnostic accuracy and cost-effectiveness of a handheld ultrasound device (HUD) for cardiac evaluation during consultation sessions in noncardiology settings.

**Methods and results:**

The study comprised 139 patients (mean age 68 ± 17 years, 52% male) referred for SE after cardiology consultation in noncardiology departments. Before transport to echo lab, all patients were scanned with the HUD by the consulting cardiologist, and it was noted whether the clinical question was answered. The following parameters were assessed: left ventricular (LV) and right ventricular (RV) size and function, significant valvular heart disease (VHD), pericardial effusion, and inferior vena cava (IVC) size and collapse. A cost-minimization analysis was conducted to compare the cost of SE referral vs. HUD-first strategy from the hospital’s perspective. In 118 (85%) patients, it was possible to successfully answer the clinical question using the HUD. There was excellent agreement for the detection of abnormal LV size and function (*k* = 0.964), abnormal RV size and function (*k* = 0.943), pericardial effusion (*k* = 0.953), and abnormal IVC size and collapse (*k* = 0.941). There was good agreement for the detection of significant VHD (k = 0.706). The HUD-first approach led to 74% reduction in echo-related costs.

**Conclusion:**

This study demonstrates that HUDs could be used by experts as a cost-saving, first-line approach for cardiac evaluation in noncardiology inpatient settings, with high diagnostic accuracy. These findings support the selective adoption of a HUD-first strategy in consultative cardiology practice, when examinations are performed by trained cardiologists and integrated within a structured diagnostic pathway.

**Clinical problem:**

Many patients who are hospitalized for conditions unrelated to heart disease still require evaluation by a heart specialist. Traditionally, this process involves performing a comprehensive heart ultrasound test. However, these tests often take significant time, require moving patients to different areas within the hospital, increase length of stay, and can be expensive. As a result, healthcare providers are actively seeking quicker and simpler methods to assess heart health directly at the bedside.

**Study Overview:**

This study explored an alternative approach utilizing a compact, handheld ultrasound device with advanced capabilities. With this technology, a heart specialist can conduct an ultrasound examination immediately during a patient consultation, right at the bedside. The research compared the effectiveness of this rapid bedside exam with the standard, comprehensive heart ultrasound, focusing on how well each approach could address the most critical clinical questions.

**Key Findings:**

The results showed that the handheld ultrasound exam provided doctors with the necessary information in most cases. Specifically, it was highly accurate in evaluating heart pumping function, detecting fluid around the heart, and volume status. For numerous heart conditions, the findings from the handheld device closely matched those from the full cardiac ultrasound test. Introducing this device as the initial step in evaluation also led to a reduction in the number of comprehensive ultrasound tests required, resulting in significant cost savings and more efficient patient care, since the clinical question could be answered at the bedside.

**Conclusion:**

Overall, the study demonstrates that modern handheld ultrasound devices with advanced features can enable healthcare professionals to assess hospitalized patients more rapidly and effectively. This approach has the potential to enhance patient comfort, decrease waiting times, and lower healthcare costs, all while providing doctors with reliable information to support clinical decision making.

## Graphical Abstract

**Graphical Abstract:**
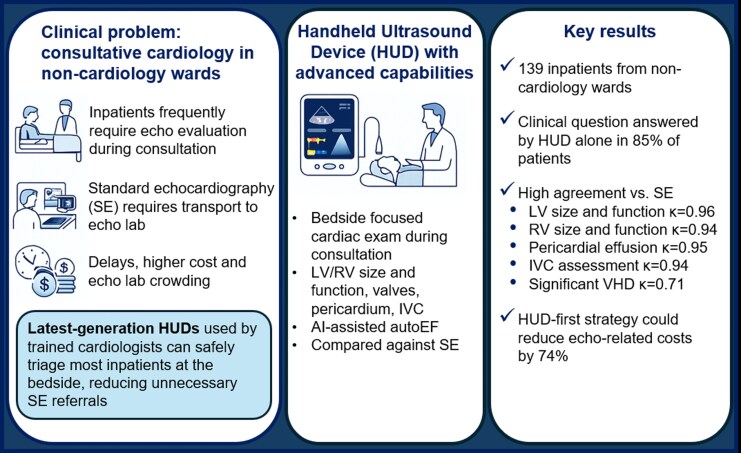

## Introduction

During consultative evaluations in noncardiology settings of busy tertiary care hospitals, cardiologists often encounter clinical questions that cannot be adequately addressed through physical examination alone, such as the assessment of pericardial effusion or evaluation of left and/or right ventricular systolic function. Echocardiography is the cornerstone of cardiovascular imaging and is superior to physical examination for the diagnosis of heart disease.^1,2^ Hospitalized patients are frequently referred for a comprehensive echocardiographic examination due to signs or symptoms suggestive of cardiovascular disease. Transporting inpatients to the echocardiography laboratory may result in patient discomfort, increased healthcare costs, delays in examination scheduling, and overcrowding of the echocardiography facility. Conversely, the use of cart-based standard echocardiography (SE) systems outside the laboratory is often impractical due to logistical challenges and time constraints, thereby limiting their utility for cardiological evaluations in inpatient settings. The use of ultraportable handheld ultrasound devices (HUDs) with high diagnostic accuracy and low cost at the patient’s bedside has the potential to significantly influence daily clinical practice.^3,4^ Previous studies using HUDs in various patient populations have shown that, in most cases, handheld ultrasound provided clinicians with timely and valuable diagnostic information, often obviating the need for further evaluation in the echocardiography laboratory.^5–10^ Newer generation HUDs are equipped with M-mode, colour and spectral Doppler capabilities, alongside improved 2D grey-scale image quality.^11,12^ We hypothesized that the use of a HUD during consultative cardiology evaluations in noncardiology settings could reduce the number of referrals for SE exams, decrease associated healthcare costs, and expedite clinical workflow for hospitalized patients; however, data on the clinical safety and diagnostic accuracy of such approach are still limited. Importantly, the present study focused on the use of HUDs within the framework of consultative cardiology, whereby cardiac imaging is performed by trained cardiologists at the request of noncardiology services, rather than as point-of-care ultrasound (POCUS) performed by noncardiologists as part of initial bedside assessment. This distinction is critical, as consultative echocardiography differs fundamentally from POCUS in terms of operator expertise, diagnostic scope, and implications for clinical decision making and downstream testing.

The purpose of the present study was to evaluate the clinical utility, diagnostic accuracy, and cost-effectiveness of an HUD with enhanced imaging capabilities, as compared with current standard practice in consultative cardiology assessments.

## Methods

### Patient population

This prospective study comprised consecutive, nonselected, adult patients hospitalized in noncardiology departments of a tertiary hospital, for whom a cardiology evaluation was requested by their treating physicians. Patients were eligible for inclusion if the consulting cardiologist deemed an echocardiographic evaluation necessary. Enrolment occurred over a 4-month period. All patients provided verbal informed consent and were recorded in the institution’s single-centre echocardiography database. All treatment decisions were based on standard practice, i.e. information provided by SE machines, thus there was no additional risk to patient safety. The study received proper ethical approval from the scientific board of Papageorgiou General Hospital and the Research Ethics Committee of the Aristotle University of Thessaloniki (Ref. No. 332 487/2023) and conformed to the principles of the Declaration of Helsinki for biomedical research.

### Study design

The study involved two clinical cardiologists, each holding Level II accreditation in echocardiography, who routinely conduct consultative cardiology assessments as part of their standard clinical responsibilities and were experienced in the use of the HUD that was employed in the study. For each patient requiring cardiology consultation, the cardiologist performed a thorough clinical assessment and if deemed necessary after physical examination the patient was further referred for a transthoracic echocardiogram. Patients were subsequently scheduled for an SE examination at the earliest available opportunity based on the consulting cardiologist’s clinical recommendation on the level of urgency and were independently examined on the same day by the consulting cardiologist using the HUD, prior to undergoing the SE. The findings of the two imaging methods were then compared regarding the presence of cardiac abnormalities. Additionally, the study assessed whether using the ultraportable device as a screening tool during consultations—referred to as the ‘HUD-first strategy’—could help reduce unnecessary referrals and lower healthcare costs compared to the conventional approach of referring all patients for a full echocardiography exam. The time intervals between the request for an echocardiographic examination and the final cardiac report for both the HUD and the SE exams were also compared. Although the findings of the HUD examination informed the consulting cardiologist’s hypothetical referral decision, all patients underwent SE irrespective of HUD findings, according to the study protocol. This design allowed unbiased validation of HUD diagnostic performance against standard echocardiography as the reference method, while simultaneously enabling evaluation of a hypothetical HUD-first triage strategy for consultative cardiology.

### Cardiac evaluation using HUD

The HUD (Kosmos, Echonous Inc., Redmond, WA, USA) used in this study consists of an 8-inch handheld tablet (dimensions: 146 × 216 × 59 mm, weight: 657 g) and features a 2–5 MHz phased-array transducer with 2D-grey scale imaging, M-mode, colour Doppler, as well as pulsed wave and continuous wave Doppler, and tissue doppler imaging (see Supplementary data online, *Figure S1*). It can also automatically calculate the left ventricular (LV) ejection fraction using artificial intelligence-enabled algorithm within 15 s.^13^ The device permits automatic storage of the images without using an ECG signal. All the images were saved on the device and transferred after each session to a workstation for archiving purposes. A focused exam was performed at bedside with the patient in the left lateral decubitus position if possible. The focused echocardiography assessment was obtained using any of the available features of the Kosmos HUD at the discretion of the consulting cardiologists, who were formally trained and accredited in echocardiography. After each scan, the cardiologist was asked to answer specific questions (with a yes/no response) regarding the presence of cardiac abnormalities. In summary, the following primary outcomes were assessed: (i) LV size and function, (ii) right ventricular (RV) size and function, (iii) significant (more than moderate) valvular heart disease (VHD), (iv) pericardial effusion, (v) inferior vena cava (IVC) size and collapse, and (vi) other noteworthy abnormal findings as secondary outcomes (thrombus/mass presence, LV hypertrophy, LA dilation, aortic root dilation, pleural effusion). The overall image quality of the HUD study was recorded and categorized as ‘good,’ ‘moderate,’ ‘poor,’ or ‘non-diagnostic’. A good quality study was defined by a good endocardial border delineation (less than two myocardial segments not optimally visualized) and good valve visualization with optimal visualization of all the cardiac anatomy. The images were classified as moderate quality when there was suboptimal endocardial border delineation with two to six myocardial segments not optimally visualized or the cardiac valves were not well visualized in more than one view. The images were graded as poor when there was suboptimal endocardial border delineation with suboptimal visualization of more than six myocardial segments and/or the cardiac valves were not well visualized in all the views. The images were defined as nondiagnostic when the ventricles and/or the cardiac valves were not visualized in any of the views.

For each patient, the consulting cardiologist noted whether the findings of the HUD were adequate to address the clinical question, whether a further comprehensive echocardiographic study was necessary and the level of urgency (either during the current hospitalization or later on outpatient basis), as well as the time duration required to complete the focused HUD examination.

### Cardiac evaluation by standard echocardiography

As part of the study protocol, all patients underwent a SE examination with a conventional cart-based ultrasound system (Epiq, Philips, Inc.) in the echocardiography laboratory, in accordance with international guidelines.^14^ These examinations were performed by an independent echocardiography consultant with a Level III accreditation in echocardiography, who was blinded to the findings of the HUD study. The SE study served as the reference method for comparison and validation of the HUD findings. In cases where discrepancies between the two modalities were present, clinical decision-making and patient management were guided by the results of the SE examination. The image quality of the SE examination was recorded and categorized as ‘good,’ ‘moderate,’ ‘poor,’ or ‘non-diagnostic’ using the same classification system applied to the HUD studies. For each patient, the consulting cardiologist noted whether the findings of the SE exam were sufficient to answer the clinical question or whether further diagnostic imaging was necessary. In our institution, the duration allocated for acquisition and reporting of a comprehensive echocardiographic study is standardized at 40 min per examination.

### Health economic evaluation

A cost-minimization analysis was conducted from the hospital’s perspective to compare the total costs of the standard practice of direct referral for a comprehensive echocardiographic examination vs. the HUD-first approach, in which the ultraportable device is used as an initial screening tool during the cardiology consultation. The time horizon for the analysis was limited to the duration of hospitalization, and therefore no discounting was applied. The total cost for each strategy included staff-related expenses, equipment acquisition and maintenance costs, hospital operational costs, patient transport time to the echocardiography laboratory, and the average time required for image acquisition and reporting. The analysis focused on identifying cost differences between the two strategies, assuming diagnostic adequacy sufficient to guide in-hospital clinical decision making, rather than equivalence in downstream clinical outcomes, which were not assessed in this study. Given that this was a cost-minimization analysis based on directly observed hospital costs over a short time horizon, formal sensitivity or probabilistic analyses were not performed. However, potential uncertainty was limited as cost inputs were directly measured from hospital records, and clinical outcomes were assumed equivalent.

### Statistical analysis

Normality of data distribution was assessed prior to analysis. Continuous variables were expressed as means ± standard deviation or medians with interquartile range (IQR) when not normally distributed; categorical variables were presented as counts and/or percentages. Comparison between continuous variables was performed using paired Student’s *t*-test or analysis of variance (ANOVA) with Bonferroni’s correction in post-hoc tests, whereas the variables not normally distributed were compared with the non-parametric Wilcoxon signed ranks test. Categorical variables were compared using the chi-square test for independent samples and the McNemar test for dichotomous dependent variables between two related groups. Agreement between the HUD and SE in detecting pathological findings was evaluated using weighted Cohen’s kappa coefficient.^15^ Kappa coefficient is a statistical measure of interrater agreement for qualitative items. The strength of agreement was interpreted using standard benchmarks: <0 no agreement, 0–0.20 slight agreement, 0.21–0.40 fair agreement, 0.41–0.60 moderate agreement, 0.61–0.80 substantial agreement, 0.81–1.00 almost perfect agreement. Kappa values were given with their 95% confidence intervals (CIs). The sensitivity, specificity, positive predictive value, negative predictive value, and overall diagnostic accuracy of the Kosmos HUD in detecting pathological findings were calculated using 2 × 2 tables, and the corresponding 95% CIs were determined. Statistical significance was defined as a two-tailed *P*-value <0.05, and the type II error rate (β) was set at 0.20. All statistical analyses were conducted using SPSS software, version 22.0 (IBM Corp., Chicago, IL, USA).

#### Power calculation

A power analysis was performed to determine the sample size required to detect a significant reduction in the number of SE referrals when using a HUD-first strategy. Based on previous literature, we estimated that approximately 70% of patients evaluated with the HUD during consultation would not require a subsequent SE examination. Using McNemar’s test for paired proportions, a total sample size of 130 patients would provide greater than 99% power to detect this difference at a two-tailed significance level of α = 0.05. This sample size was therefore deemed sufficient to evaluate the primary outcome of the study.

## Results

### Study population

The study prospectively enrolled 139 consecutive patients (mean age 68 ± 17 years, 52% male, BMI 27.1 ± 8.0 kg/m^2^) who were hospitalized in noncardiology departments of our hospital and were referred for echocardiographic evaluation following cardiology consultation. The study population comprised patients admitted to the following departments: internal medicine (*n* = 39), oncology/haematology (*n* = 27), neurology (*n* = 15), general surgery (*n* = 15), urology (*n* = 10), obstetrics/gynaecology (*n* = 8), nephrology (*n* = 6), vascular surgery (*n* = 5), orthopaedics (*n* = 5), cardiothoracic surgery (=3), dermatology/plastic surgery (*n* = 3), and other (*n* = 3). The primary clinical indications prompting cardiac evaluation by echocardiogram are listed in *Table 1*.

**Table 1:** Clinical requests for cardiology consultation in noncardiology departments

	Patients (%)
Preoperative evaluation	23 (16.5)
Assessment of LV function/HF	22 (15.8)
Stroke	18 (12.9)
Chemotherapy-related oncology referrals	14 (10.1)
Dyspnoea/chest pain	13 (9.4)
Elevated cardiac enzymes	8 (5.8)
Fever/suspected IE	8 (5.8)
Patient’s volume status	7 (5.1)
Syncope	5 (3.4)
Tachycardia	5 (3.4)
Pericardial effusion	5 (3.4)
RV function/pulmonary embolism	4 (2.9)
Other	7 (5.1)

IE: infectious endocarditis, HF: heart failure, LV: left ventricle.

### Diagnostic accuracy

The prevalence of abnormal echocardiographic findings in the study population, as detected by both the HUD and SE, is presented in *Table 2* and was not significantly different between the two methods. In total, there were 72 (52%) patients in this population with various cardiac abnormalities identified by standard echocardiography. The HUD demonstrated high diagnostic performance in detecting patients with any cardiac abnormality, with a sensitivity of 94% (95% CI 0.85 to 0.98), specificity of 96% (95% CI 0.88 to 0.99), positive predictive value of 95% (95% CI 0.87 to 0.98), negative predictive value of 94% (95% CI 0.87 to 0.98), and total accuracy 95% (95% CI 0.90 to 0.98). When analysed separately, the HUD demonstrated high diagnostic performance for major echocardiographic domains. For detection of abnormal LV size and/or systolic function, sensitivity was 100% (95% CI 0.78 to 0.99), specificity 99% (95% CI 0.95 to 1.00), and overall accuracy 99% (95% CI 0.96 to 1.00). Corresponding values for RV size and/or function were 90% (95% CI 0.56 to 1.00), 100% (95% CI 0.97 to 1.00), and 99% (95% CI 0.96 to 1.00), respectively. Diagnostic performance for significant VHD was lower, with sensitivity 71% (95% CI 0.49 to 0.87), specificity 96% (95% CI 0.91 to 0.99), and overall accuracy 92% (95% CI 0.86 to 0.96), reflecting the focused nature of HUD examinations and the complexity of valvular severity grading.

**Table 2:** Prevalence of abnormal findings in the study population (*n* = 139) as detected by standard echocardiography and the HUD

	SE *n* (%)	HUD *n* (%)	*P*-value
Abnormal LV size and systolic function	15 (10.9)	16 (11.6)	1.000
Abnormal RV size and function	10 (7.2)	9 (6.5)	1.000
Significant valvular heart disease	24 (17.9)	21 (15.7)	0.549
Pericardial effusion	12 (8.7)	11 (8.0)	1.000
Abnormal IVC size and collapse	20 (14.6)	20 (14.6)	1.000
LV hypertrophy	8 (5.8)	8 (5.8)	1.000
LA dilation	15 (10.8)	15 (10.8)	1.000
Dilated aortic root	5 (3.6)	5 (3.6)	1.000
Mass/thrombus	3 (2.2)	3 (2.2)	1.000
Pleural effusion	5 (3.6)	5 (3.6)	1.000

IVC: inferior vena cava, LA: left atrium, LV: left ventricle, NS: nonsignificant, RV: right ventricle, SE: standard echocardiography.

**Figure 1:**
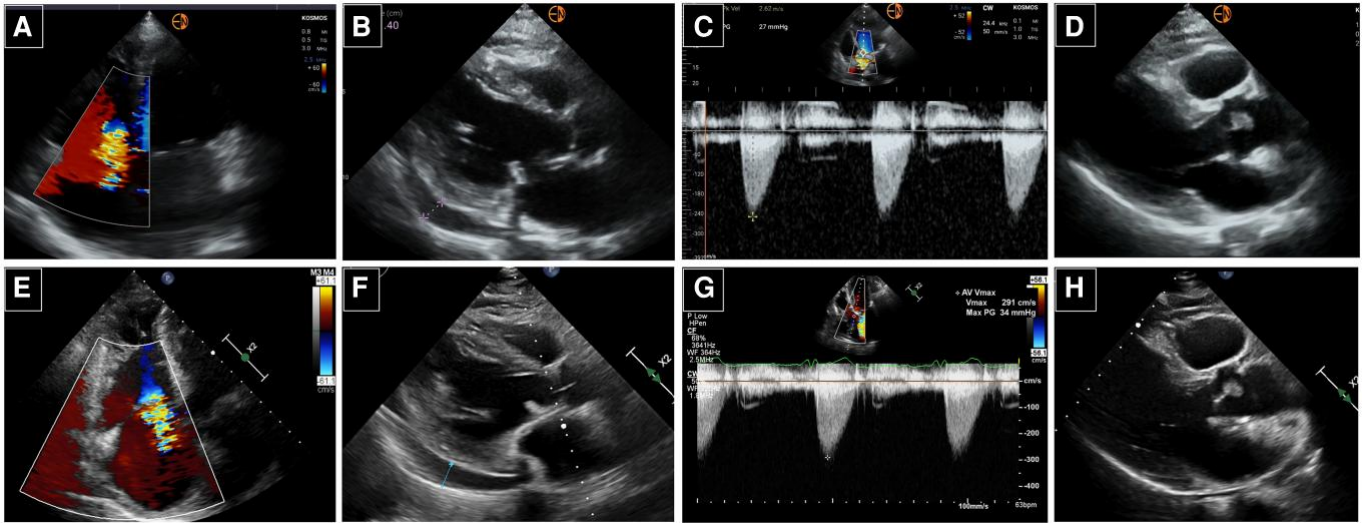
Apical four-chamber view obtained using HUD (*A*) and SE (*E*) in a patient with severe tricuspid regurgitation. Parasternal long-axis view obtained using HUD (*B*) and SE (*F*) in a patient with moderate pericardial effusion. Aortic peak jet velocity measured with CW Doppler on five-chamber view using HUD (*C*) and SE (*G*) in a patient with mild aortic stenosis. Parasternal long-axis view obtained using HUD (*D*) and SE (*H*) in a patient with papillary fibroelastoma.

The overall agreement between the HUD and the SE examinations for identifying cardiac abnormalities was excellent (*k* = 0.897, 95% CI 0.822 to 0.969). There was excellent agreement for the detection of abnormal LV size and function (*k* = 0.964, 95% CI 0.893 to 1.000), abnormal RV size and function (*k* = 0.943, 95% CI 0.833 to 1.000), pericardial effusion (*k* = 0.953, 95% CI 0.860 to 1.000), abnormal IVC size and collapse (*k* = 0.941, 95%CI 0.861 to 1.000), LV hypertrophy (*k* = 1.0, 95% CI 1.000 to 1.000), and LA dilatation (*k* = 0.925, 95% CI 0.822 to 1.000). There was good agreement for the detection of significant VHD (*k* = 0.706, 95% CI 0.544 to 0.869). Percent agreement between HUD and standard echocardiography was also calculated to complement kappa statistics, particularly for findings with low prevalence. Percent agreement exceeded 95% for LV size and function, RV size and function, pericardial effusion, inferior vena cava assessment, and LV hypertrophy, and was 91% for significant valvular heart disease. Representative images of patients scanned with both methods are shown in *Figure 1*.

Discrepancies between HUD and SE for significant VHD primarily involved grading of severity, particularly in cases of tricuspid and mitral valve disease, rather than failure to detect the presence of valvular pathology. No cases of severe valvular disease requiring urgent management were missed by HUD examination (*Table 3*).

**Table 3:** Misclassified valvular lesions (*n* = 11)

Standard echocardiography	HUD
Aortic stenosis	
Mild (*n* = 1)	Moderate
Moderate (*n* = 1)	Mild
Severe (*n* = 0)	—
Mitral regurgitation	
Mild (*n* = 1)	Moderate
Moderate (*n* = 4)	Mild
Severe (*n* = 0)	—
Tricuspid regurgitation	
Mild (*n* = 2)	Moderate
Moderate (*n* = 2)	Mild
Severe (*n* = 0)	—

**Figure 2:**
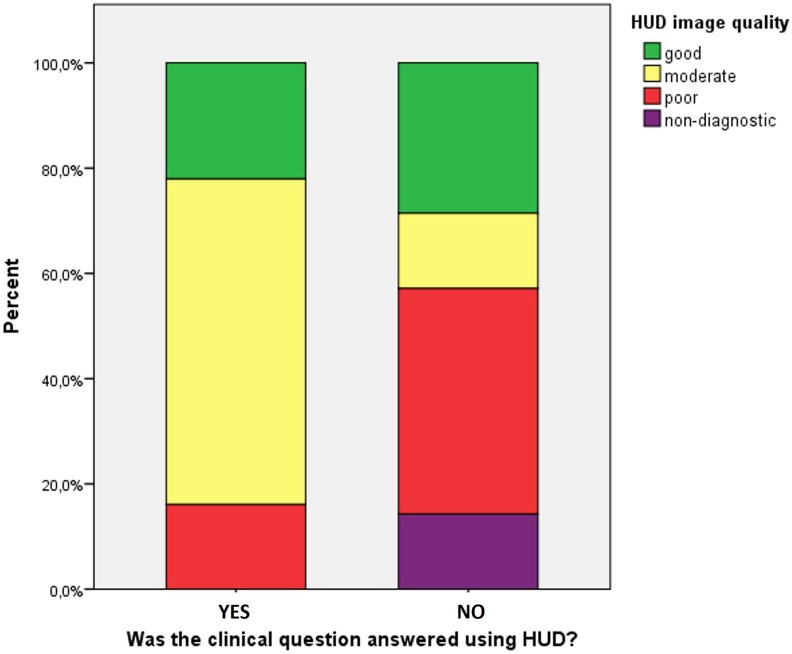
Bar graph illustrating the relationship between HUD image quality (categorized as good, moderate, poor, or nondiagnostic) and the ability to address the clinical question. Most scans with good or moderate image quality were associated with successful clinical assessments, whereas poor and nondiagnostic images were more frequently associated with unanswered clinical questions.

**Figure 3:**
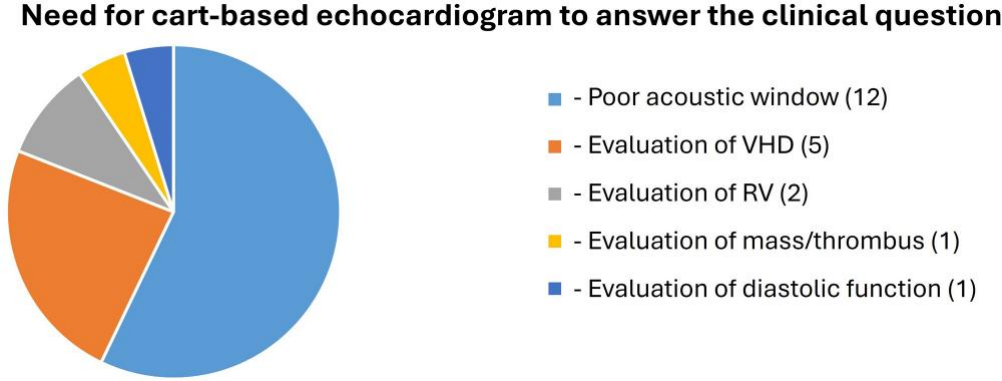
Pie chart demonstrating the primary reasons for which the comprehensive echocardiographic study was deemed necessary to address the clinical question.

In 118 (85%) patients, it was possible to successfully answer the clinical question by the HUD examination alone. Based on the findings of the HUD cardiac assessment, the consulting cardiologist would recommend a comprehensive echocardiogram during hospitalization for 21 (15%) patients, and on an outpatient basis for an additional 24 (17%) patients. The overall image quality of SE acquisitions was visually assessed as good in 40% of cases, moderate in 50%, and poor in 10%. For the HUD examinations, image quality was graded as good in 23% of cases, moderate in 55%, poor in 20%, and nondiagnostic in 2%. In a post-hoc analysis, the percentage of nondiagnostic and poor image quality HUD studies was significantly lower among patients in whom the clinical question was successfully answered, compared to those in whom it was not (16.1% vs. 57.2%, *P* < 0.001), *Figure 2* The primary reasons for which the comprehensive SE study was deemed necessary to address the clinical question are presented in *Figure 3*. The mean duration of image acquisition using the HUD during consultation sessions was 5.7 ± 2.2 min and the mean reporting time using a prespecified form was estimated at approximately 5 min. In contrast, the average waiting time between the request for an SE examination and the final cardiac report was 2 days.

Eventually, for 120 out of 139 patients the SE study did not yield substantial additional information for clinical management beyond that which was already provided by the HUD examination. The additional information obtained from SE included detailed valvular assessment in 13 patients, exclusion of cardiac source of emboli in 2 patients, evaluation of RV size and function in 1 patient, assessment of regional wall motion abnormalities in 1 patient, detailed evaluation of a mass on the tricuspid valve in 1 patient, and 1 normal SE exam. Importantly, 18 of these 19 patients were referred for a complete SE examination after the clinical assessment and the initial HUD exam by the consulting cardiologist (7 patients during hospitalization and 11 on an outpatient basis); the 1 patient who was not referred had a moderate tricuspid regurgitation. Moreover, in 7 cases the clinical question remained unresolved even after the SE examination, and further cardiac imaging was needed (5 transoesophageal echocardiograms, 1 contrast-enhanced echocardiogram, and 1 CT pulmonary angiography). Overall, the combined HUD-first strategy—incorporating HUD evaluation during cardiology consultation prior to potential referral for SE—would have successfully addressed the clinical question in 94.9% of patients, comparable to the conventional strategy of referring all patients for full echocardiographic examination.

### Cost-effectiveness analysis results

The cost associated with the standard clinical practice of referring patients to the echocardiography laboratory was calculated on a per-scan basis. The mean annual number of echocardiographic examinations performed following cardiology consultation requests was estimated to be approximately 1000. The purchase cost of the cart-based SE machines used in this study was approximately €159,000, with a 10-year equipment depreciation estimated at €15 900 per year. The cost per comprehensive SE examination was calculated at €4 462 euros. This figure included the cost of the echocardiography consultant’s time for image acquisition and reporting, the cost of inpatient transportation from the ward to the echocardiography laboratory, equipment-related costs (purchase and maintenance), and other associated hospital expenses.

The echocardiographic examination using the HUD was considered an adjunct to the physical examination and therefore not charged separately. However, for the purpose of the economic analysis, we incorporated in the final cost the initial purchase cost of the Kosmos HUD, which was approximately €9,000, with an annual depreciation rate of €900. Thus, the cost per HUD examination was estimated at €5.60 per patient, accounting for equipment-related costs and the additional time spent by the consulting cardiologist.

Applying these data to our study results, the total cost of performing 139 comprehensive SE examinations under the current clinical practice was €6202. In contrast, using the HUD-first strategy, the total cost was €1,598, as further investigation with SE was required in only 21 patients. This corresponds to a 74% cost reduction in echocardiography-related expenses. Extrapolated to an average of 1000 patients annually, the implementation of a HUD-based screening strategy during cardiology consultations in noncardiology settings could yield projected annual savings of approximately €33 465 in our institution. Extrapolation to settings with higher consultant and hospital staff hourly rates, as well as greater volumes of cardiac evaluations in noncardiology inpatient settings, suggests substantially higher potential cost savings.

## Discussion

This prospective study supports the role of a contemporary HUD as a diagnostic gatekeeper during consultative cardiology evaluations in noncardiology inpatient settings, enabling accurate triage of patients who require comprehensive echocardiography while safely reducing unnecessary referrals. Our findings indicate that in 85% of this targeted patient population, the HUD provided the consulting cardiologist with sufficient information to immediately answer the clinical question during the consultation session, without the need for further evaluation with SE exam during hospitalization. Importantly, all clinically relevant findings missed or clarified by SE occurred in patients whom the consulting cardiologist had already identified for further SE after HUD assessment. These results support the potential of modern HUDs to serve as first-line diagnostic tools in inpatient consultation settings, offering a clinically robust, time-efficient, and economically sustainable alternative to unselected routine referral to the echocardiography laboratory.

The diagnostic accuracy of the HUD in this study was excellent across a wide spectrum of clinically relevant findings. Agreement with SE was particularly strong for the assessment of ventricular size and function, pericardial effusion, and IVC evaluation, consistent with previous studies validating the use of HUDs in both inpatient and outpatient settings.^2,5^ In the present cohort, 85% of clinical questions could be confidently answered using HUD alone at patient bedside and almost 1 out of 2 patients (48%) in this population did not have notable cardiac abnormalities. These results align with former studies demonstrating that portable ultrasound devices can provide significant clinical information during consultation rounds, frequently obviating the need for further diagnostic testing and expediting clinical decision making.^7,10^ Noteworthy, the portable ultrasound device employed in these older studies was considerably larger (weighing approximately 2.4 kg) and lacked the portability of newer generation pocket-sized HUDs used in our study. Although image quality was lower for HUD studies compared to SE, the high rate of diagnostic adequacy underscores the utility of focused cardiac imaging in targeted clinical scenarios, where the objective is to promptly and effectively address specific clinical questions rather than to perform comprehensive cardiac imaging.

Importantly, in most previous investigations, the necessity for a complete echocardiographic study was mainly driven by the need for haemodynamic assessment by Doppler imaging, since earlier devices lacked spectral Doppler or high-quality colour Doppler capabilities. Our study extends these earlier findings into a contemporary context, using a HUD equipped with both spectral and colour Doppler capabilities; this advancement is particularly relevant for VHD assessment. Although diagnostic agreement for significant VHD was lower than for other parameters, it remained clinically acceptable. Recent studies have highlighted the importance of continuous-wave Doppler capability for accurate quantification of aortic stenosis severity and other significant valvular abnormalities—a well-recognized limitation of earlier-generation HUDs.^11,12,16,17^ Our findings are in keeping with these observations; although the HUD examination effectively triaged patients requiring further evaluation, there was still a subset of patients, especially those with complex VHD, that SE was required. These findings emphasize that HUD should not be viewed as a substitute for comprehensive echocardiography in the definitive grading of complex valvular heart disease, but rather as an effective triage tool to identify patients who require full Doppler-based evaluation.

The utility of HUD in preoperative cardiac evaluation has also been demonstrated in randomized studies, with handheld echocardiography providing additional information beyond clinical assessment and improving decision-making in surgical candidates.^18^ In our population, 16.5% of patients were surgical patients referred for preoperative echocardiographic evaluation. Our results extend this rationale to a broader inpatient population, supporting HUD integration not only in preoperative evaluation but also across a wide range of clinical scenarios within both medical and surgical services.

In addition to demonstrating high diagnostic performance, this study illustrates the significant cost-effectiveness of a HUD-first approach integrated into consultative cardiology practice. An estimated 74% reduction in echocardiography-related expenses was achieved using a HUD-first strategy in this study. This degree of cost reduction is substantially greater than that reported in earlier economic analyses of first-generation portable ultrasound systems, in which demonstrated savings between 29% to 35%,^9,10,19,20^ and similar to findings from studies evaluating newer-generation HUDs.^6^ It is important to note that the reported cost savings may underestimate the full economic benefit to the hospital. The cost-minimization analysis did not fully account for the broader economic and organizational impact—particularly the delay typically observed between the request for a full echocardiographic examination and the delivery of the final cardiac report. An additional advantage of the HUD-first strategy relates to workflow efficiency. Use of a structured, focused reporting template enabled rapid documentation of findings for less complex cases, substantially reducing reporting delays compared with conventional echocardiography workflows and facilitating timely clinical decision-making. Overall, these findings are consistent with the growing emphasis on value-based care in contemporary health systems.

### Clinical implications and future directions

This study was designed to evaluate the feasibility of integrating HUDs into routine clinical practice, as a first-line diagnostic approach in noncardiology settings. Τhe previously established distinction between cardiac POCUS and consultative echocardiography has been based primarily on the role in patient management of the healthcare professional performing the exam, namely, examinations conducted by the treating clinician vs. those performed by a consulting team at the request of the treating clinician.^21^ The proposed model is not opportunistic scanning during ward rounds, but rather a structured consultative cardiology workflow, where the referring team requests cardiology input and the cardiologist after physical examination determines need for echocardiographic examination and can use the HUD to decide further referral to echo lab (SE yes or no) and the level of emergency (SE immediately or on outpatient basis). With advances in image quality, Doppler capabilities, and AI-assisted quantitative analysis on the horizon, the diagnostic performance of HUDs is expected to improve further, allowing for consultative cardiology evaluations to be performed without the routine necessity for a full comprehensive echocardiogram. Such a shift has the potential to facilitate timely referrals of patients for further diagnostic tests, prompt modifications in treatment strategies, and significantly impact the workflow of cardiology services, alleviate overcrowding in echocardiography laboratories, and optimize resource utilization across hospital departments. Nevertheless, the importance of appropriate operator training and accreditation in echocardiography needs to be emphasized; in this study, all HUD studies were performed by experienced cardiologists with formal echocardiographic training. The generalizability of these findings to non-expert users remains uncertain and requires further investigation and curriculum development; it is not supported by the results of the present study.

### Limitations

This was a single-centre study conducted at a tertiary care hospital, potentially limiting external generalizability of the findings. Moreover, we did not evaluate clinical outcomes or the impact of HUD-guided decision making on length of hospitalization stay, morbidity, or mortality. Future randomized studies comparing a HUD-guided strategy to routine clinical practice will be necessary to determine the effect of HUD implementation on patient outcomes. It should be acknowledged that Cohen’s kappa values may be inflated in settings with low prevalence of certain findings. For this reason, percent agreement is also reported and should be interpreted alongside kappa statistics when evaluating agreement for infrequent abnormalities such as pericardial effusion or intracardiac masses. Although the study was adequately powered to detect a reduction in standard echocardiography referrals, it was not powered to assess diagnostic accuracy for individual echocardiographic findings. Accordingly, analyses of specific parameters should be interpreted as exploratory. It is also crucial to acknowledge that all HUD cardiac examinations in this study were performed and interpreted by cardiology consultants with many years of training and experience in transthoracic echocardiography. Thus, the results are not directly generalizable to operators with less training and limited experience in echocardiography; adequate training in echocardiography is essential for the acquisition, interpretation, and clinical integration of HUD findings.

## Conclusions

In summary, this study demonstrates that contemporary HUDs could be used as a cost-effective, first-line approach for cardiac evaluation in noncardiology inpatient settings, achieving high diagnostic accuracy. When operated by trained cardiologists, information obtained from HUDs allows for the efficient triage of patients, enabling most clinical questions to be addressed at the bedside without the need for comprehensive echocardiography, while accurately identifying those requiring further diagnostic evaluation. These findings support the selective adoption of a HUD-first strategy in consultative cardiology practice, when examinations are performed by trained cardiologists and integrated within a structured diagnostic pathway.

## Supplementary Material

qyag047_Supplementary_Data

## Data Availability

The data underlying this article will be shared upon reasonable request to the corresponding author.

## Lead author biography

Dr Stella-Lida Papadopoulou is a cardiologist with a scientific interest in multimodality cardiovascular imaging and digital cardiology. She obtained her MD from Aristotle University of Thessaloniki (Greece). She was a clinical research fellow at the departments of Cardiology and Radiology at Erasmus Medical Center (Rotterdam, NL). Her work resulted in her PhD thesis ‘Imaging of coronary atherosclerosis with CT coronary angiography’ in 2016. After completing her cardiology training in 2020, she received a scholarship from the Hellenic Society of Cardiology for postgraduate fellowship in Digital Cardiology at Papageorgiou General Hospital and subsequently she specialized in advanced echocardiography. Currently, she is a consultant cardiologist at Papageorgiou General Hospital in Thessaloniki, Greece.
