# Supplementary figures and images for: Diagnostic accuracy and cost-effectiveness of a handheld ultrasound device for cardiac evaluation in noncardiology settings
Source: Eur Heart J Imaging Methods Pract. 2026 Mar 13;4(1):qyag047. doi: 10.1093/ehjimp/qyag047 (PMC13020536; doi:10.1093/ehjimp/qyag047)

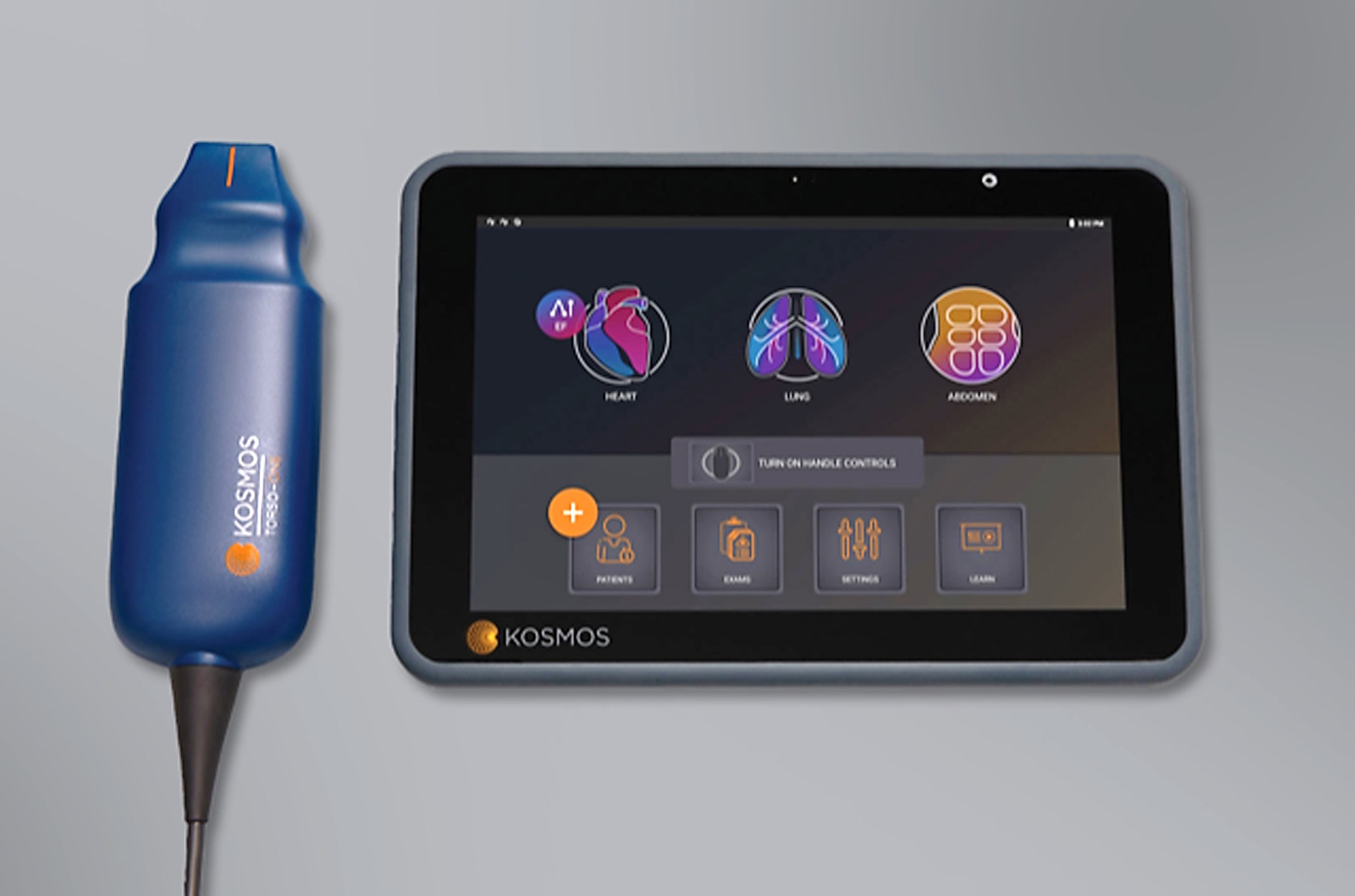

Supplement: qyag047_Supplementary_Data [file qyag047_supplementary_data.jpeg]
